# Supplementary material for: Experimental Morogoro Virus Infection in Its Natural Host, Mastomys natalensis
Source: Viruses. 2021 May 7;13(5):851. doi: 10.3390/v13050851 (PMC8151005; doi:10.3390/v13050851)
Supplement: Supplementary file 1 [file viruses-13-00851-s001.zip › viruses-1187527-supplementary.pdf]

Table S1: Master mix set-up for the qRT-PCR.

Reagent	Concentration per reaction	Volume per reaction [µl]
SuperScript™ III Reverse Transcriptase/ Platinum™ Taq DNA Polymerase Mix	0,5 µl	0,5
Superscript mix [2x]	1x	12,5
Probe [10 µM]	0,2 µM	0,5
Forward Primer [10 µM]	0,5 µM	1,25
Reverse Primer [10 µM]	0,52 µM	1,3
Nuclease-free H_2O		3,95
Template		5
Total volume		25

Table S2. Cycling parameters for qRT-PCRs with the Platinum™ Taq DNA Polymerase Mix.

Step	Temperature [°C]	Time
RT-step	55	20 min
Polymerase Activation	95	2 min
Cycling (45x)	95	15 sec
	55	45 sec
	72	15 sec

Table S3. Viral titers in kidney, lung and gonads of infected *M. natalensis* following inoculation or exposure to MORV or MOBV.

Group	weeks post-birth	kidney pos/tested	kidney FFU/organ min	kidney FFU/organ max	lung pos/tested	lung FFU/organ min	lung FFU/organ max	gonads pos/tested	gonads FFU/organ min	gonads FFU/organ max
MORV inoculation day 2 (*n* = 5)	1	1/1	2,60E+06		1/1	1,15E+08		n.t.		
	2	1/1	8,75E+04		1/1	2,14E+05		n.t.		
	20-22	1/3	1,35E+04		2/3	7,69E+02	2,71E+04	2/2	1,22E+03	1,84E+03
MORV inoculation day 6 (*n* = 15)	2	1/4	1,43E+03		4/4	1,25E+03	8,00E+04	2/4	5,00E+02	8,75E+02
	3	4/4	3,75E+03	1,00E+05	4/4	2,13E+05	4,38E+05	2/2	9,29E+04	4,50E+05
	4	3/4	2,00E+02	2,50E+03	3/4	2,00E+04	6,25E+04	1/2	3,89E+04	
	5	1/3	2,00E+03		2/3	5,00E+01	2,00E+04	1/3	7,50E+02	
MORV inoculation day 14 (*n* = 7)	3	2/4	1,00E+03	7,00E+04	1/4	4,17E+02		n.t.		
	4	0/3	negative		0/3	negative		0/3	negative	
MORV inoculation day 27 (*n* = 4)	5	0/2	negative		0/2	negative		0/2	negative	
	8	0/2	negative		0/2	negative		0/1	negative	
MORV exposure from birth (*n* = 34)	0-1	4/12	2,50E+02	6,00E+04	4/12	1,00E+03	3,00E+05	n.t.		
	2-3	1/8	1,11E+02		4/8	2,19E+01	1,00E+04	1/2	1,67E+01	
	4-5	0/4	negative		0/4	negative		0/4	negative	
	8-10	4/5	2,09E+04	1,98E+05	4/5	2,62E+03	1,28E+05	3/4	1,08E+03	3,39E+04
	16-20	2/5	2,41E+03	1,48E+04	1/5	3,75E+04		2/5	2,22E+02	1,09E+03
MORV exposure from day 25 (*n* = 3)	8	0/3	negative		0/3	negative		0/3	negative	
MOBV inoculation day 2 (*n* = 4)	1	0/1	negative		0/1	negative		n.t.		
	2	1/1	9,17E+03		1/1	9,09E+02		n.t.		
	10	0/2	negative		0/2	negative		0/2	negative	

n.t. = not tested.

Table S4. Viral titers in spleen, liver, heart and brain of infected *M. natalensis* following inoculation or exposure to MORV or MOBV.

Group	weeks post-birth	spleen pos/tested	spleen FFU/organ min	spleen FFU/organ max	liver pos/tested	liver FFU/organ min	liver FFU/organ max	heart pos/tested	heart FFU/organ min	heart FFU/organ max	brain pos/tested	brain FFU/organ min	brain FFU/organ max
MORV inoculation day 2 (n = 5)	1	1/1	1,10E+05		1/1	7,92E+03		1/1	7,50E+05		1/1	4,42E+02	
	2	1/1	2,33E+02		1/1	3,26E+02		1/1	6,43E+04		1/1	4,52E+04	
	20-22	0/3	negative		1/3	2,27E+01		1/3	2,08E+02		1/3	4,17E+02	
MORV inoculation day 6 (n = 15)	2	0/4	negative		0/4	negative		2/4	1,00E+03	3,33E+03	1/4	3,33E+02	
	3	4/4	1,67E+03	2,67E+04	0/4	negative		4/4	2,50E+03	9,17E+03	4/4	1,25E+03	3,15E+04
	4	3/4	9,09E+02	3,57E+03	1/4	4,17E+02		3/4	1,11E+03	1,00E+04	3/4	1,39E+02	1,18E+03
	5	1/3	1,33E+03		0/3	negative		1/3	3,85E+01		0/3	negative	
MORV inoculation day 14 (n = 7)	3	1/4	1,67E+04		1/4	2,65E+03		2/4	7,14E+03	9,09E+03	2/4	1,04E+03	1,13E+04
	4	0/3	negative		0/3	negative		0/3	negative		0/3	negative	
MORV inoculation day 27 (n = 4)	5	0/2	negative		0/2	negative		0/2	negative		0/2	negative	
	8	0/2	negative		0/2	negative		0/2	negative		0/2	negative	
MORV exposure from birth (n = 34)	0-1	2/10	1,50E+03	5,00E+03	1/12	1,00E+03		3/8	5,00E+02	1,00E+04	3/10	5,00E+01	1,11E+03
	2-3	2/8	4,17E+01	6,25E+01	1/8	6,25E+01		0/8	negative		3/8	1,67E+01	5,00E+03
	4-5	0/4	negative		0/4	negative		0/4	negative		0/4	negative	
	8-10	3/5	3,70E+02	6,16E+04	3/5	4,81E+02	2,44E+03	3/3	1,07E+03	2,63E+03	4/5	1,68E+03	3,55E+04
	16-20	2/5	1,39E+02	6,00E+03	1/5	6,82E+03		1/5	3,67E+03		1/5	9,26E+02	
MORV exposure from day 25 (n = 3)	8	0/3	negative		0/3	negative		n.t.			0/3	negative	
MOBV inoculation day 2 (n = 4)	1	0/1	negative		0/1	negative		0/1	negative		0/1	negative	
	2	0/1	negative		0/1	negative		1/1	1,50E+05		1/1	3,70E+04	
	10	0/2	negative		0/2	negative		n.t.			0/2	negative	

n.t. = not tested.

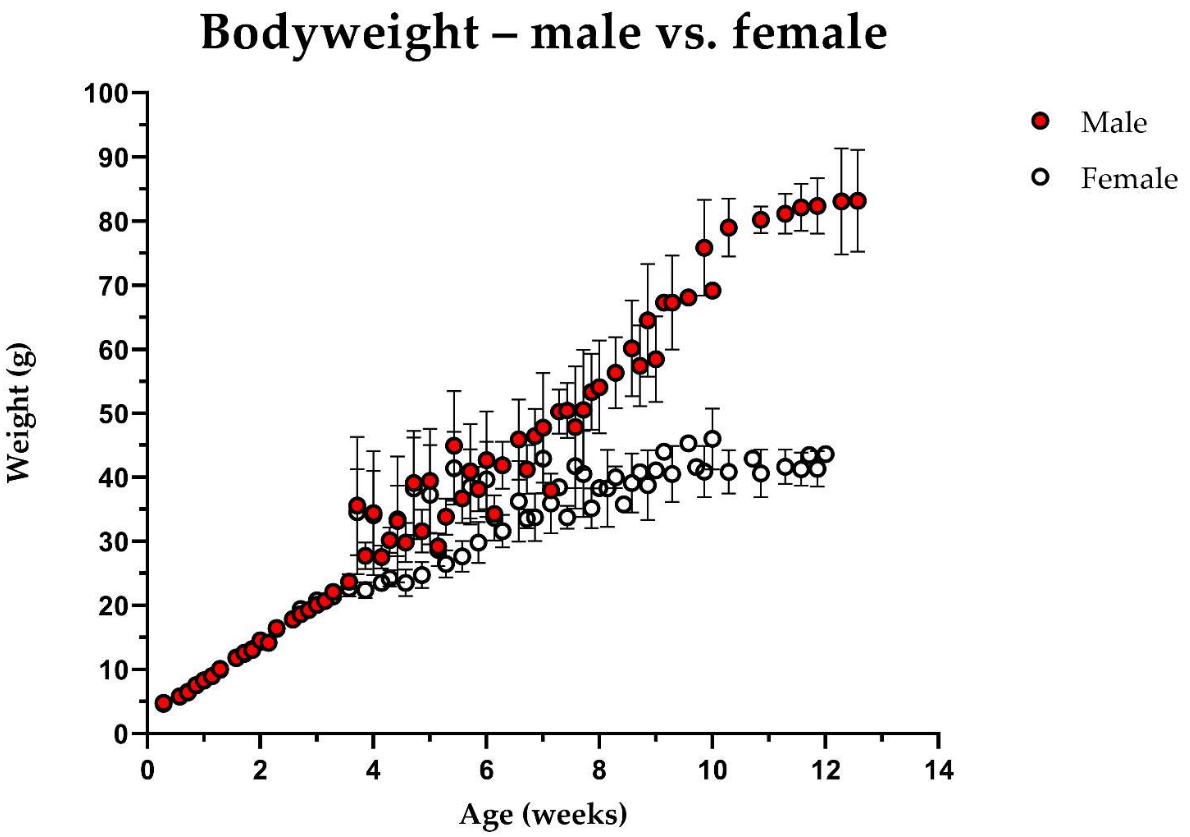

Figure S1. Growth of male and female Mastomys. The body weight of *M. natalensis* (n = 51) during the first 14 weeks of life is shown as mean with standard deviation. Males (n = 26) are depicted as red dots and females (n = 25) as white dots.
